# New Record of the Biting Midge *Leptoconops noei* in Northern Spain: Notes on Its Seasonal Abundance and Flying Height Preference

**DOI:** 10.1673/031.013.4501

**Published:** 2013-05-31

**Authors:** Mikel A. González, Sergio López, Arturo Goldarazena

**Affiliations:** 1 NEIKER-TECNALIA, Basque Institute of Agricultural Research and Development, Entomology and Virology Laboratory, 46 01080, Vitoria, Spain.

**Keywords:** bites, breeding sites, Ceratopogonidae, dry ice traps, first record, seasonal populations, sticky traps

## Abstract

During the summers of 2004–2006, harmful outbreaks of *Leptoconops noei* Clastrier and Coluzzi (Diptera: Ceratopogonidae) occurred in a small region in the southern part of Alava (Basque Country, Spain). Two types of traps were placed for monitoring *L. noei*: CDC traps baited with dry ice in eight different locations and sticky traps at three different heights (two, four, and six meters). A total of 1,823 adults were captured with dry ice traps and 163 specimens with sticky papers. Dry-baited collections occurred between June and August in two of the eight samplings places. Significant differences were observed concerning the vertical distribution of *L. noei*. The most specimens were captured at a height of two meters. A specific area near the riverside composed of sandy matter was described as the main developmental site for *L. noei*. This is the first record of *L. noei* in Spain.

## Introduction

The Ceratopogonidae is a large and diverse family belonging to the order Diptera with more than 110 genera and 6,089 living species (Borkent 2012). Only four genera are considered as blood-sucking flies in this family: *Culicoides, Leptoconops, Forcipomyia* (subgenus *Lasiohelea*), and *Austroconops* (only present in the Australian region) (Ronderos et al. 2003). They feed on a great number of vertebrate hosts such as humans, mammals, birds, reptiles, amphibians, and also insects (Delécolle 1985). Their importance is owing to their capacity to transmit pathogens of medical importance (e.g., nematodes, viruses, bacteria, protozoa, and helminths), whereas the genus *Leptoconops* is known in various parts of the world because of its nuisance and upset bites during the day. Furthermore, *Leptoconops* can cause serious injuries, especially when they are present in great numbers (Carrieri et al. 2007). *Leptoconops* midges affect tourist resorts and therefore cause serious economic losses and health problems (Majori and Bettini 1971; Strickman et al. 1995). The immediate effects of *Leptoconops* spp. bites are considered less severe than the subsequent lesions that can result; usually, papules remain intensively itchy for months and sometimes can became infected, resulting in major problems (Whitsel and Schoeppner 1966; Aussel 1993).

In Europe, 11 species of *Leptoconops* have been reported, six belonging to the subgenus *Holoconops* and five to the subgenus *Leptoconops* (Szadziewski and Borkent 2004). The genus *Leptoconops* is poorly known in Spain, and only few data exist about their ecology, seasonal populations, and biting habits. Only two species have been reported in Spain: *Leptoconops* (*Leptoconops) bezzii* (Noé) and *Leptoconops (Holoconops) kerteszi* Kieffer 1908 (Delecolle 1999). In France, nine species of *Leptoconops sensu lato* are present, therefore it is likely that there remain undiscovered species in Spain.

This work comes as a result of massive biting episodes in humans that occurred in a small village, where several people were hospitalized complaining of painful bites (Lopez de Lacalle 2004). The biting midges appeared on the first fortnight of June and remained there until August.

The seasonal abundance and vertical distribution of *Leptoconops noei* (Diptera: Ceratopogonidae) are described for first time in the Iberian Peninsula during 2005 and 2006 respectively. Furthermore, photographs and notes about their identification are provided.

## Materials and Methods

The study was carried out in the locality of Santa Cruz de Campezo (Alava, Basque Country, Spain) (latitude 42° 40′ 21.19″, longitude 2° 21′ 52.88″, 570 m.a.s.l.). This village is situated in a valley surrounded by high mountains, where the principal geographic resources are plots, field camps, small woods, farms, and the Ega river bordering the northern side of the village. According to the Euskalmet (Basque Meteorology Office), it belongs to a submediterranean climatic area with warm temperature, dry summers, and moderated annual rainfalls. The average precipitation is 950.4 mm per year.

**Figure 1.**
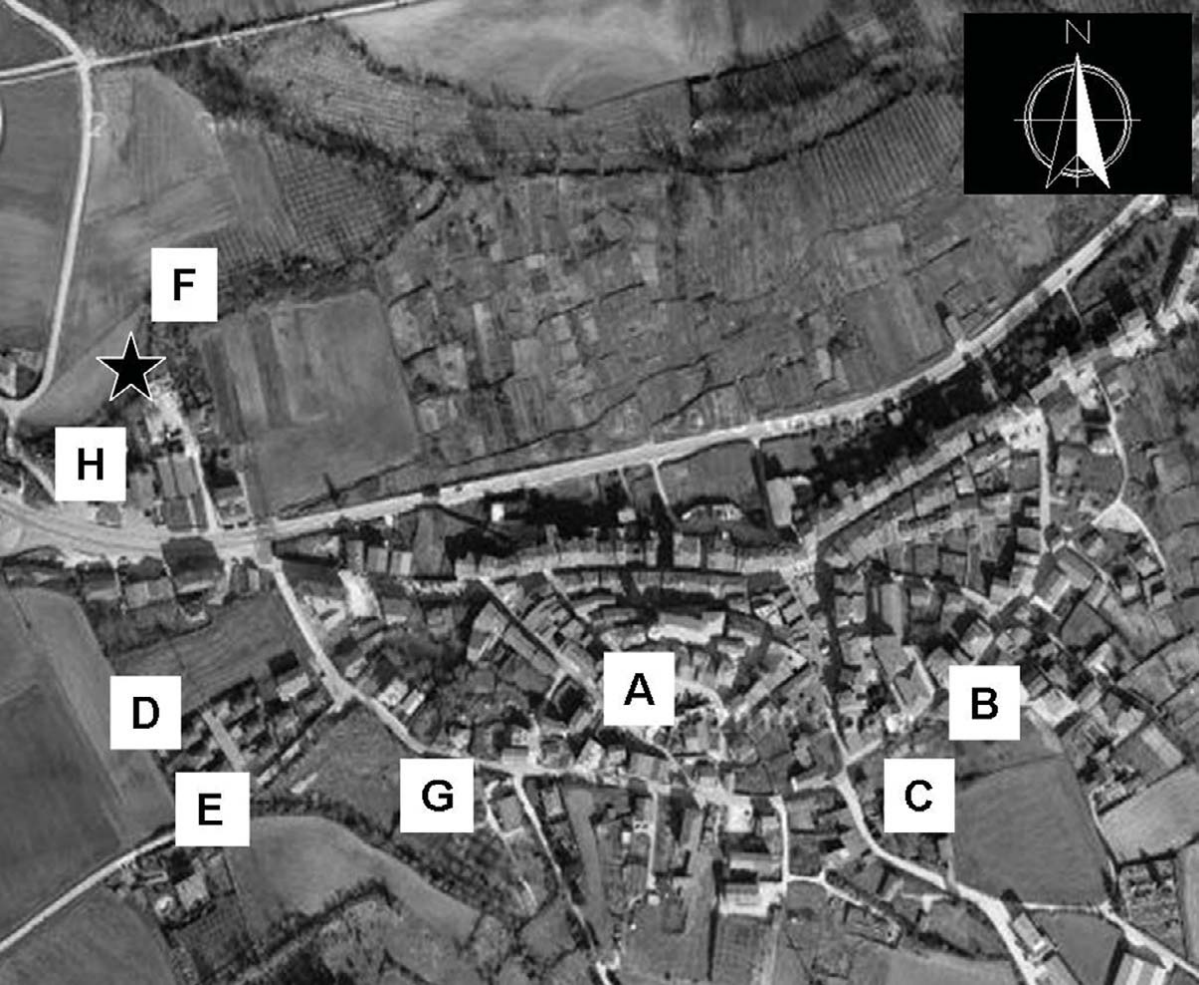
Study area showing the eight different sampling places in Santa Cruz de Campezo (Alava, north Spain). The black star indicates the place where the *Leptoconops noei* specimens emerged from the river. Letters correspond with the descriptions in the text. High quality figures are available online.

Eight CDC traps (Entomopraxis, G852, model 1212, http://www.entomopraxis.com/) without a light source and equipped with a dry-ice bucket with holes above it were placed in eight putative sites in a randomized design (every 15 days the traps were rotated) (Figure 1): a) in the village square, b) near a sheep farm, c) near a duck farm, d and e) one on each side near manure composed of feces of cows, sheep, and ducks, f) near the river, g) near a chicken farm, h) in the swimming pool near the river. Every five days, carbon dioxide was replaced and fortnight plastic jars were collected and transported to the laboratory. The traps worked from January to December of 2005.

Another field assay was performed from 1 June 2006 to 31 July 2006. This assay consisted of placing sticky-trap papers covered with castor oil (20 × 20 cm) in posts at heights of two, four, and six meters in those places where *Leptoconops* midges had been captured in great numbers the previous year. Specimens were removed, putting a drop of a solvent (Goo-Gone, Homax Products, Inc., www.homaxproducts.com) on each insect. Samples were collected every fortnight.

Identifications of all specimens of *L. noei* were made by using two different methods. Adults from CO_2_ collections were preserved in 70% ethanol and then mounted in Hoyer's medium for identification under a Leica DM500B microscope compound using the appropriate taxonomical keys (Clastrier and Coluzzi 1973). Targeted specimens captured with sticky traps were examined under a Leica MZ95 stereoscopic microscope to separate them from other species of insects. All the photographs were taken with a Leica DFC300 camera.

Larval breeding sites were obtained from small samplings (10 cm^3^) taken with a flat trowel in sandy areas near the riverside. Larvae were extracted with flotation techniques under laboratory conditions (Chaker 1983). Home-made emergence traps placed on the riverside allowed the collection of a few newly-adult midges.Data of *Leptoconops* midges collections in sticky traps were analyzed with Kruskal-Wallis non-parametric test followed by Dunnet's test at a significance level of α = 0.05 (SPSS 2004).

## Results

**Figure 2.**
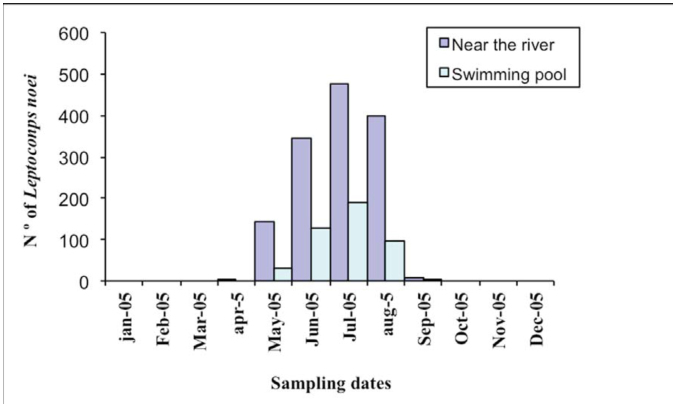
Adult collections of *Leptoconops noei* with dry-ice CDC traps during the year 2005 in two different locations: near the river (dark blue bars) and the swimming pool (light blue bars). High quality figures are available online.

A total of 1,823 adults of *L. noei* were collected during a year-long monitoring program, most being collected between May and August. *L. noei* specimens were captured in two specific sites: 1,374 adults (394 males; 980 females) near the river and 449 adults (99 males, 350 females) in the swimming pool (Figure 2). The relative sex ratio was 0.37:1 male: female (male/female × 100).

In regards to the traps at different heights, 103 specimens were collected from traps at a height of two meters, 48 from traps at a height of four meters, and 12 from traps at a height of 6 meters. According to statistical analysis, significant differences (*p* = 0.0073) were observed among the trapping at three different heights (Figure 4). Several immature stages, especially third and fourth instars of *L. noei* midges, were encountered in the first few cm of the sandy matter.

## Discussion

*L. noei* is recorded from the Iberian Peninsula for the first time. Scarce geographical distribution data about *Leptoconops* species in Europe are available except data about *Leptoconops* fossil species, which were allegedly very common in the Cretaceous period (Szadziewski 2008). Living *Leptoconops* species were recorded by Delécolle (1999), who indicated the presence of *L. bezzii* and *L. kerteszi* in the Monegros region (north-eastern Spain), attributing to them an exclusive Mediterranean distribution.

*Leptoconops* species are not commonly collected in light traps due to their diurnal activity. They are generally attracted with dry ice, simulating animals' breathing. Other techniques commonly used are mechanical aspirator-trapping and chromotropic traps (Raspi et al. 2007), sticky traps (Carrieri et al. 2007), or human volunteers (Perich et al. 1995; Strickman et al. 1995).

**Figure 3.**
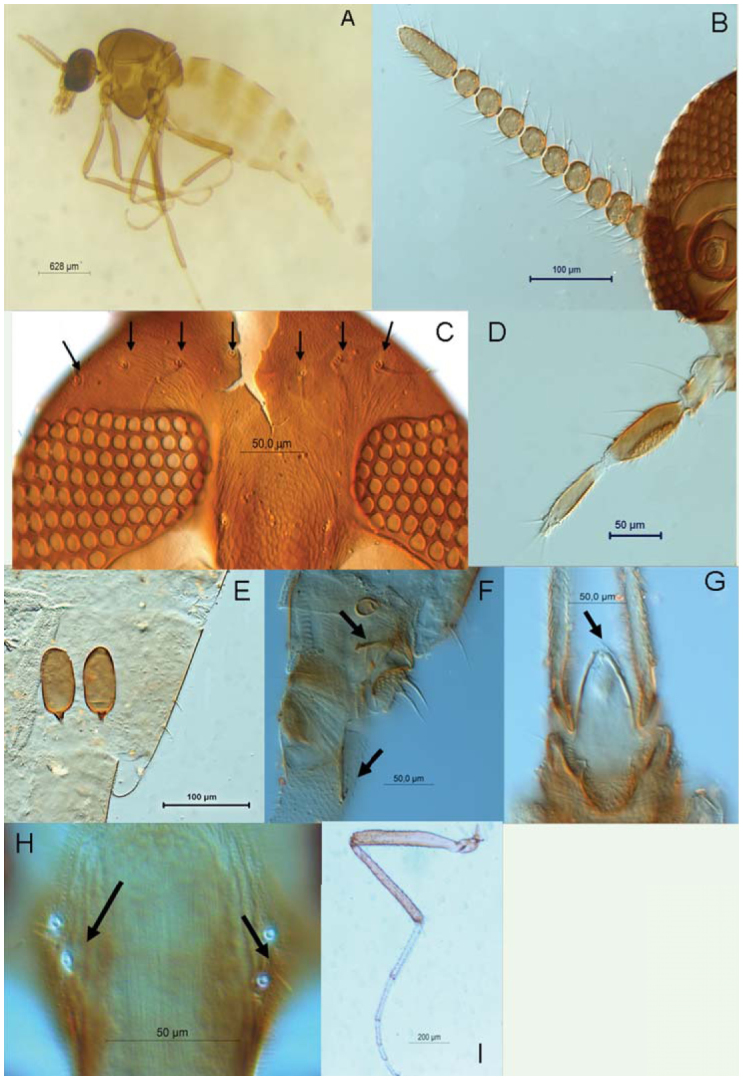
*Leptoconops noei*. A) General aspect of a female, B) Antennal segments, C) Vertex setae (black arrows), D) Palpus, E) Spermathecae, F) Genitalia, upper arrow showing the arm of the preatrial plaque ventral and below arrow indicating the armature genital in lateral view, G) Anal cone, arrow showing the armature of the anal cone in ventral view, H) Supra-orbital setae, I) Foreleg. High quality figures are available online.

Collected midges were unequivocally assigned to the subgenus *Leptoconops sensu stricto*, according to their 14-segmented antennae (Clastrier and Coluzzi 1973) and their palpus with several pits (De Meillon and Wirth 1991). This is in contrast to the subgenus *Holoconops*, which bears 13-segmented antennae and a palpus with a deep enclosed sensory pit (Raspi et al. 2007). *L. noei* is characterized by its hyaline wings without hairs, widely separated eyes in both sexes, well-developed fronts, 13 flagellar-segments in males, and 12 flagellar-segments in females. Its palpus bears a characteristic fusion of segments four and five, appearing four-segmented. The sensory pit is confined to a large, open depression on the third segment showing several external sensillas. The spermathecaes are sausage-shaped. In females, the anal cercus is large, elongate, and cone-shaped. The male's gonocoxite is approximated at the base; the gonostylus ends in an apical socketed peg, unique in Ceratopogonidae (De Meillon and Wirth 1991). See characters in Figure 3 about *L. noei*.

Seasonal abundances of *L. noei were* partially similar to other studies with another species, in which it was reported two flying periods for *Leptoconops kerteszi* per year in Italy: from April to June and from September to November (Raspi et al. 2007). In contrast, our results with *L. noei* showed captures from May to August, similar to *L. irritons* in Italy, which exhibits only one flight period (Bettini et al. 1969; Raspi et al. 2007). This univoltine pattern of flight could be caused by the seasonal river's water-level, which would cover the sandy sides of the river and thus make breeding impossible.

Carrieri et al. (2007) observed the vertical distribution of *L. noei* and *L. irritons* in vegetated areas and in open areas. They captured significantly more biting midges at two meters in vegetated areas rather than at four and six meters, but similar collections at two and four meters were recorded in comparison to six meters in open areas. Considering the trees and shrubby vegetation act as a natural barrier to flying, specimens are more likely to be obtained at lower heights. In addition to this likelihood, it is well known that some members in the family Ceratopogonidae, especially *Culicoides* species, show height preferences, likely associated with their vertebrate-host presence as well as other factors (Braverman and Linley 1993; Venter et al. 2009). For this reason, it is possible that *Leptoconops* midges were collected in great numbers at heights below three meters because their vertebratehosts were active near the ground level. However, it is necessary to take into consideration the influence of other environmental factors such as vegetation distribution, light sources, weather conditions, and relative humidity (Venter et al. 2009). The presence of bird-feeding *Culicoides* species have been documented at 26 meters above the ground level (Braverman and Linley 1993).

During 2004 (one year before the study), *L. noei* bites on humans were registered up to two km away from the river breeding places, causing more than 50 people to be hospitalized. One year after, adults were exclusively captured in two places near the river. Long-distance dispersal is common in small insects such us *Culicoides* species, which can be transported by the wind (10–40 km/h) up to 700 km in certain conditions (Sellers 1992), but their spreading is poor under no-wind conditions. Recently, balloon-supported nets at 170–200 meters above ground level were used by Sanders et al. (2011) to demonstrate the presence of *Culicoides* over the United Kingdom. This research explains the high potential importance of the wind in the dispersal of some insect species. Metereological data about wind, speed, altitude, and trajectory have been used to support the bluetongue virus (S-1) outbreak in the Basque Country in 2007–2008. It was proposed that the most likely scenario was the arrival of BTV infected *Culicoides* midges from warm air masses from the south of the Iberian Peninsula (García-Lastra et al. 2012). However, in this study (2005–2006), the wind conditions did not seem to play any role in the dispersal of *Leptoconops* midges, because they were encountered only in two of the eight traps near the riversides (with no more than 200 meters distance between the traps). Most likely, the poor dispersion was a consequence of low or zero wind speeds (Sellers 1992).

The genus *Leptoconops* is distributed over wide areas and is relatively widespread in wetlands with sandy or silty-clay soils, especially in swampy coastal areas and in ponds of salt water (Kettle 1962). They can be found in sandy beaches (Khadri et al. 2002; Rohani et al. 2006), sand covered by seaweed and other sea drift in California (Brenner and Wargo 1972), moist sandy soils devoid of vegetation near the sea, saltwater ponds, and along the banks of rivers in Italy (Raspi et al. 2007). The larvae are quite similar to *Culicoides* species, but *Leptoconops* head capsule possesses well-developed posteriorly directed apodemes extending into the prothorax, and the abdomen is secondarily divided (Borkent and Spinelli 2007).

Biting midges can be controlled by using chemical products or biological control measures, but the first control attempts have produced unsuccessful results because of the midges' high tolerance level to adulticides and to their small size (1–3 mm length), which allows them to penetrate most mosquito screening and netting. Protective clothing and the use of repellents are the most costeffective means of relief for humans entering habitats of biting midges during peak activity periods (Perich et al. 1995). Larvae control is problematic due to the location of the midges' breeding sites, i.e., near rivers, beaches, great sandy areas, or tourist sites (Carrieri et al. 2007).

The use of powerful and effective repellents in serious cases of infestation is recommended. Ears have been reported as one of the most preferred places for *Leptoconops* midges to bite (Perich et al. 1995), but they have also been reported to bite the face and neck. Repellents such as diethyl methylbenzamide (DEET), 1-(3-Cyclohexen-1-yl-carbonyl)-2-methylpiperidine (AI3-37220), (2-hydroxymethylcyclohexyl) acetic acid lactone (CIC-4), and 1-(3-cyclohexen-1-ylcarbonyl)-piperidine (AI3-35765) have been tested in humans against *Leptoconops americanus* and obtained effective results (Perich et al. 1995).

The results of this study about biological aspects of *Leptoconops* midges are novel, and further studies are necessary to know the presence of these species in other parts of the Iberian Peninsula. Unfortunately, they were not collected in the bluetongue surveillance monitoring programs run with CDC light traps in Spain, because they are not commonly attracted by light source traps. Additionally, there is a lack of knowledge about the midges in human populations because their bites sometimes go unnoticed.

**Figure 4.**
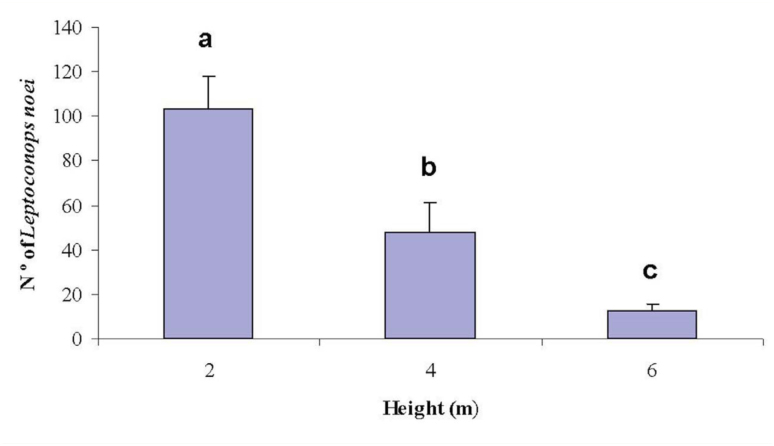
Catches (Mean ± S.E) of *Leptoconops noei* at three different heights (two, four, and six meters). Different letters mean significant differences at a significance level of α = 0.05 (Kruskal- Wallis followed by Dunnett's test). High quality figures are available online.
